# Push-out bond strength of intra-orifice barrier materials: Bulk-fill composite versus calcium silicate cement

**DOI:** 10.15171/joddd.2018.002

**Published:** 2018-03-14

**Authors:** Taha Özyurek, Gülşah Uslu, Koray Yilmaz

**Affiliations:** ^1^Ondokuz Mayis University, Faculty of Dentistry, Department of Endodontics, Samsun, Turkey; ^2^Çorum Oral and Dental Health Hospital Çorum, Turkey

**Keywords:** Bulk-fill Composite, Calcium-silicate, Push-out

## Abstract

***Background.*** The aim of this study was to compare the push-out bond strengths of calcium silicate-based ProRoot MTA and Biodentine cements and SureFil SDR and EverX Posterior bulk-fill composite resins.

***Methods.*** Twenty-four single-rooted maxillary central incisors were sectioned below the cementoenamel junction, and the root canals were instrumented using rotary files. Thereafter, a parallel post drill was used to obtain a standardized root canal dimension. The roots were randomly assigned to one of the following groups with respect to the intra-orifice barrier used: ProRoot MTA; Biodentine; SureFil SDR; EverX Posterior. Five 1-mm-thick sections were obtained from the coronal aspect of each root. Push-out bond strength testing was performed and data were analyzed with Kruskal-Wallis and post hoc Dunn tests (P<0.05).

***Results.*** SureFil SDR and EverX Posterior bulk-fill composite resins’ bond strengths were significantly higher than ProRoot MTA and Biodentine calcium silicate cements. However, no statistically significant differences were observed between bulk-fill composite resins values and calcium silicate cement values.

***Conclusion.*** Within the limitations of present study, calcium silicate-based ProRoot MTA cement’s push-out bond strength was lower than those of Biodentine, SureFil SDR and EverX Posterior materials.

## Introduction

Coronal leakage is one of the most important reasons for the failure after root canal treatment.^1^ Ray and Trope^2^ reported that the quality of coronal restoration is more important in protecting the periapical health than the quality of root canal filling. For this purpose, it has been suggested that to prevent the penetration of oral fluids and microorganisms into the root canals 3‒4 mm of coronal gutta-percha should be removed from the root canal and an intra-orifice barrier should be placed at canal orifice^3^ or a pulpal base should be placed using a restorative material.^4^ Previous studies have reported that covering the pulpal base with intra-orifice barrier materials after the root canal treatment constructs a secondary defense line against bacterial leakage.^4,5^ For this purpose, different materials have been employed, including temporary filling materials, glass-ionomer cement, composite resin, MTA and IRM.^6,7^

Today, MTA is also safely used in conservative pulpal treatments, root resorption treatments, and apexification procedures.^8,9^ MTA has been shown to be a bioactive material inducing the formation of hard tissues; it is well-tolerated by the tissues it contacts because of its biocompatibili.y^10,11^ However, long setting time of MTA (4‒6 hours) and the difficulties in adjusting the consistency while mixing make this material non-practical for clinical use.^12^ Numerous materials based on tri-calcium silicate have been developed and introduced to the market in order to eliminate these disadvantages of MTA.^13-14^ Biodentine (BD; Septodont, Saint-Maur-des-Fosses, France) is a new endodontic cement containing tri-calcium silicate and calcium carbonate, with a setting time of 12 minutes.^15^ The manufacturer claims that BD can be used as a replacement for dentin tissue for restorative purposes and as direct pulp cupping material for endodontic purposes, as well as restoration of perforations and as a root-end filling material.^16^

SureFil SDR flow (SDR; Dentsply Caulk, Milford, DE, USA), one of the bulk-fill composite resins recently introduced to the market, is a silorane-based nano- and micro-hybrid composite with low viscosity; its shrinkage stress is lower than conventional fluids.^17,18^ Another fiber-reinforced bulk-fill composite resin, also newly introduced to the market, is EverX Posterior (EXP; GC Dental Products Corp., Tokyo, Japan).

In comprehensive literature research, no study was found, comparing the push-out bond strengths of SDR and EXP bulk-fill composite resins. For this reason, the aim of the present study was to compare the push-out bond strengths of calcium silicate-based ProRoot MTA and BD cements and SDR and EXP bulk-fill composite resins. The null hypothesis of the present study was there would be no statistically significant difference between the push-out bond strengths of the tested materials.

## Methods

### Specimen Selection

Twenty-four single-rooted (0‒5°)^19^ maxillary central incisors, with no signs of calcification and extracted due to periodontal reasons, were included in the present study. The teeth were examined under ×2.5 magnification, and those with fractures or cracks or multiple apical foramina were excluded and replaced with new ones. In order to ensure the standardization, the crowns of the teeth were removed (by ensuring 16 mm of root length) with a fine diamond disc (Gebr. Brasseler GmbH & Co., Lemgo, Germany) at cementoenamel junction perpendicularly to the long axis of the teeth under water-cooling.

### Root Canal Preparation

Under ×2.5 magnification, the root canals of the teeth were penetrated using a #15 K-file (Dentsply Maillefer, Ballaigues, Switzerland); the file was inserted until it could be seen at the apical foramen, and then the working length was set at 1 mm shorter than this length. In order to shape the root canals, ProTaper Next (PTN; Dentsply Maillefer) rotary file system’s X1, X2, X3 and X4 files were used respectively. The files were used at 300 rpm and 200 g cm^-1^ torque values in DR’S CHOICE program of VDW Reciproc Gold (VDW, Munich, Germany) endodontic motor. Each of the new file sets was used for shaping 4 canals and discarded. After each file, the root canals were irrigated with 2 mL of 5.25% NaOCl solution. Then, by using a parallel post drill with a diameter of 1.25 mm (ParaPost XT, Size 5; Coltene/Whaledent, Summit County, OH, USA), 10-mm-length gaps were prepared within the root canals. For final irrigation of the root canals, 2 mL of 17% EDTA (Vista Dental Products, USA) for 2 minutes and then 2 mL of 5% NaOCl for 2 minutes and 5 mL of distilled water were utilized.

### Preparation of the Samples for Push-out Bond Strength Test

Five 1-mm-thick transverse slices were taken under water-cooling (Isomet, Buehler, Lake Bluff, IL, USA) in corono-apical direction from each tooth. The slices were randomly divided into 4 groups (n=30). In the ProRoot MTA and BD groups, the materials were prepared in accordance with the instructions of manufacturer. The materials were placed on the dentin slices on a glass slab by using a hand plugger (Dentsply Maillefer), and the residual material was removed using a plastic spatula. In the SDR and EXP groups, the canals of the dentin slices were etched for 15 seconds using 35% phosphoric acid (3M ESPE, St. Paul, MN, USA), rinsed for 15 seconds and air-dried under low-level pressure of air (left in moist form). Two-step etch-and-rinse adhesive Prime & Bond NT (Dentsply DeTrey) was applied and kept for 20 seconds, and then the canals were dried with low-level pressure of air for 5 seconds and light-cured (Elipar S10; 3M ESPE) for 10 seconds. Dentin discs were filled using SureFil SDR and EverX Posterior on the glass slab, and then light-cured for 40 seconds (Elipar S10). All the prepared samples were kept at 37°C and 100% humidity for 7 days. The particulars of the tested materials are presented in Table 1.

**Table 1:** The Composition of the Tested Materials.

**Material**	**Manufacturer**	**Type**	**Composition**
**ProRoot MTA **	Dentsply, Tulsa Dental, USA	Calcium Silicate Cement	Powder: Portland cement (75%), bismuth oxide (20%), calcium sulfate dihydrate (5%), tricalcium silicate, dicalcium silicate, tricalcium aluminate, tetracalciumaluminoferrite Liquid: distilled water
**Biodentine**	Septodent, Saint-Maur-des-Fosses, Cedex, France	Calcium Silicate Cement	Powder: tricalcium silicate, dicalcium silicate, calcium carbonate and oxide, iron oxide, and zirconium oxide Liquid: calcium chloride and hydrosoluble polymer
**SureFil SDR flow**	Dentsply, Tulsa Dental, USA	Bulk-fill Composite	Matrix composition: TEGDMA, EBADMA Inorganic filler content: 68 wt%, 44 vol%, barium borosilicate glass
**EverX Posterior**	GC EUROPE N.V., Leuven, Belgium	Bulk-fill Composite	Matrix composition: Bis-GMA, PMMA, TEGDMA Inorganic filler content: 74.2 wt%, 53.6 vol% Short E-glass berller, barium glass

*PMMA, polymethylmethacrylate; bis-GMA, bisphenol-A-glycidyldimethacrylate; TEGDMA, triethylene glycol dimethacrylate; EBADMA, ethoxylatedbisphenol-A-dimethacrylate; wt%, weight percentage; vol%, volume percentage.

### Push-out Bond Strength Test

**Figure 1:**
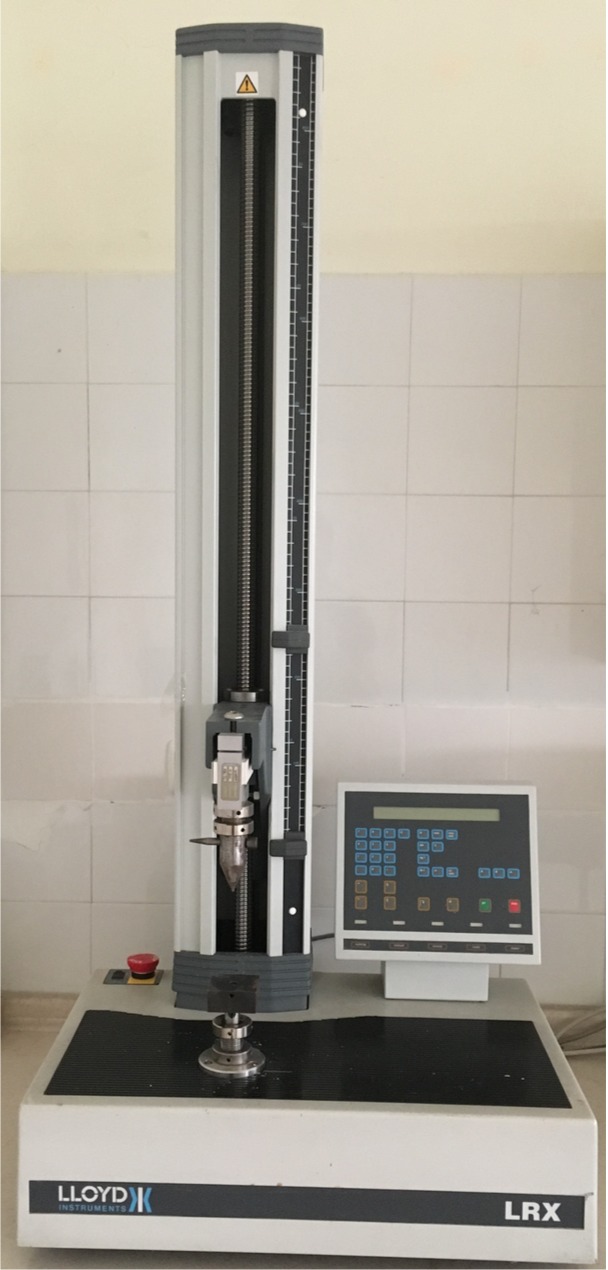
The push-out testing device.

After the samples set completely, each slice was fixed on a steel base with a hole in its center, and then connected to a universal test machine (Lloyd Instruments, Bognor Regis, England) (Figure 1). For push-out test, the stainless steel cylindrical tip with a 1-mm diameter was driven in apico-coronal direction at 1 mm/min crosshead speed until dislodgement. The Newton (N) values were converted into MPa values using the formula below:

Bond strength (MPa) = Force for dislodgement (N) / Bonded surface area (mm^2^)

Bonded surface area = 2×p×r×h (h: thickness of the dentin slice in mm; r: radius of the dentin slice canal in mm; p: constant: 3.14)

### Evaluation of Failure Patterns

**Figure 2:**
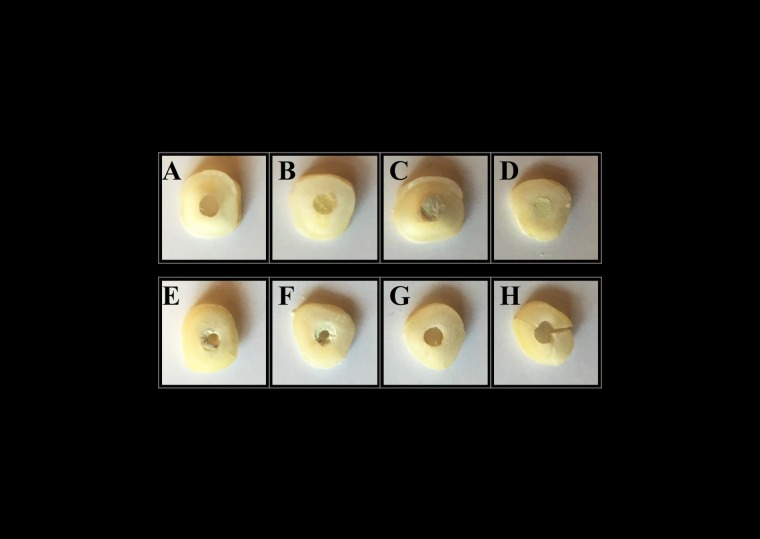
The images of the specimens before and after push-out test. (A) SDR group; (B) EverX Posterior group; (C) Biodentine group; (D) ProRoot MTA group; (E) Adhesive failure in SDR group; (F) Adhesive failure in EverX Posterior group; (G) Cohesive failure in Biodentine group; (H) Cohesive failure in ProRoot MTA group.

Following the push-out test, the slices were examined under a stereomicroscope at ×40 magnification to determine the nature of bond failure. Each sample was categorized into one of the three failure modes: adhesive failure at dentin‒material interface, cohesive failure within the material, or mixed failure, which is the combination of the two failure modes (Figure 2). The operator examining the slices was blinded to which sample matched which material.

### Statistical Analysis

The normality of data distribution was determined using Shapiro-Wilk test. Then the statistical differences between the groups were calculated using Kruskal-Wallis and post hoc Dunn tests. All the analyzes were performed using SPPS 21 (IBM-SPSS Inc., Chicago, IL, USA) software, and the statistical significance was set at 5%.

## Results

The mean and standard deviation values of the tested materials obtained from the push-out bond strength test are presented in Table 2. SDR (4.10 ± 0.88) and EXP (3.86 ± 0.72) bulk-fill composite resins’ bond strengths were significantly higher than those of ProRoot MTA (2.29 ± 0.43) and BD (3.20 ± 0.49) calcium silicate cements (P<0.05). However, there were no significant differences between bulk-fill composite resin values and calcium silicate cement values (P>0.05).

**Table 2:** Push-out bond strength values of tested intra-orifice barrier materials (MPa)

**Group**	**Mean**	**Standard Deviation**
ProRoot MTA	2.29 ^a^	0.43
Biodentine	3.20^b^	0.49
SureFil SDR	4.10^b^	0.88
EverX Posterior	3.86 ^b^	0.72
*P*-value	< .05	

^a,b^ Different superscript letters indicate a significant difference between groups (P<0.05).

The frequencies of fracture types in the test materials are presented in Table 3. No cohesive fracture was observed in SDR and EXP groups, while the mixed-type fracture was seen in the majority of cases. Mixed-type fracture was seen in 12 (40%) samples in the ProRoot MTA group and 15 (50%) samples in the BD group.

**Table 3:** Incidence of failure patterns of tested materials

	**Adhesive**	**Cohesive**	**Mixed**
**ProRoot MTA **	16 (53.3%)	2 (6.7%)	12 (40%)
**Biodentine**	14 (46.7%)	1 (3.3%)	15 (50%)
**SureFil SDR**	8 (26.7%)	0 (0%)	22 (73.3%)
**EverX Posterior**	11 (36.7%)	0 (0%)	19 (63.3%)

## Discussion

The cements used for endodontic purposes must tightly bind to the root canal walls and resist tooth movements or mechanic stresses that may occur during treatment procedures.^20-22^ For this purpose, in the present study, the push-out bond strengths of ProRoot MTA, BD, SDR and EXP materials were compared.

The bond of endodontic materials to the root dentin can be tested using different test methods such as traditional shear and push-out tests.^23^ Push-out test has been reported to be a reliable and practical test for examining the bond between materials and root dentin. In this test method, similar to the clinical environment, the fractures occur parallel to the resin‒dentin bond surface, and this method offers a better opportunity for analysis compared to the traditional shear test.^24^ For this reason, the push-out test was employed in the present study. The reason for obtaining 1-mm-thick dentin slices in the present study was that frictions that may cause misinterpretation of the results may occur in push-out test, and that it would be more reliable to utilize 1-mm-thick slices in order to minimize friction to eliminate this risk.^23-25^

According to the results of the present study, the push-out bond strength values of SDR, EXP and BD groups were significantly higher than those in the ProRoot MTA group. Therefore, the null hypothesis of the present study was rejected. Since there is no previous push-out bond strength study carried out using SDR and EXP bulk-fill composite resin in the literature, the results of the present study cannot be directly compared with other studies. However, the favorable outcomes of SDR might possibly be explained by its favorable stress behavior. In a recent study,^17^ a peculiar shrinkage behavior of the new flowable material in comparison with other flowable, nano- and micro-hybrid composites and a silorane-based material, was observed. The authors reported lower shrinkage stress, delayed point of gelation and lower shrinkage stress rates for SDR compared to the other materials that have been investigated. The high push-out bond strength of EXP material might be attributed to the ability of distribution of stresses occurring on the fibers distributed throughout the composite’s matrix.^26^

Similar to the present results, it has been reported in many studies that BD has significantly higher push-out bond strength values than ProRoot MTA.^27-30^ Moreover, Silva et al^31^ and Centenaro et al^32^ reported that BD had significantly higher push-out bond strength than MTA Angelus (Angelus, Londrina, Brazil). In another study, Alsubait et al^33^ examined the push-out bond strengths of white MTA (WMTA; ProRoot, Dentsply Maillefer), BD and Bio Aggregate (BA; Innovative Bio Ceramix, Vancouver, Canada) cements and reported no significant difference between MTA and BD. The reason for BD’s higher push-out bond strength compared to ProRoot MTA might be small molecular volume of BD cement and better penetration of cement into dentinal tubules, and consequently increased strength of bond to dentin. Moreover, because of this effect, the crystal formation might construct a dentin bridge within the dentinal tubules and thus the cement’s mechanic retention might increase.^16,34^

According to the results of present study, the fracture modes of the samples generally were mixed. The good bond of materials to dentin, because of the high push-out bond strength values exhibited by BD, SDR and EXP groups, might explain this result. Similar to the results of the present study, Centenaro et al^32^ reported higher incidence of mixed-type fracture after they examined the fracture modes of MTA Angelus and BD cements after push-out test. However, the incidence of adhesive failure has been reported to be higher in other studies.^27,35,36^ These differences in the results might be explained by differences in methodologies used in sampling and preparation.

## Conclusions

Within the limitations of the present study, it might be concluded that calcium silicate-based ProRoot MTA cement’s push-out bond strength was significantly lower than those of BD, SDR and EXP materials, with no differences between the push-out bond strength values of BD, SDR and EXP.

## Acknowledgments

None.

## Competing interests

The authors declare no competing interests with regards to the authorship and/or publication of this article.

## Ethics approval

The study protocol was approved by Ondokuz Mayis University ethics committee.
